# Department and Secretary of Public Health

**Published:** 1895-12

**Authors:** 


### DEPARTMENT AND SECRETARY OF PUBLIC HEALTH.

*To the Members of the American Medical Association:*

The American Medical Association, at its session in Washington in 1891, adopted unanimously a resolution favoring a Department and Secretary of Public Health, said secretary to be a member of the Cabinet of the President of the United States; and appointed a committee to urge upon Congress the passage of a law for the creation of such a department and secretary. This committee has been continued, with slight changes, up to this time, and have prepared and presented to Congress a suitable bill, and by such methods as were open to them, have endeavored to secure its passage. This bill is now pending in both Houses of Congress.

In the meantime, the Association has pledged itself anew at every annual meeting since 1891, and notably in the annual meeting in Baltimore, last May, to continue the work necessary to secure the desired legislation. Many State medical associations and many local medical societies have passed resolutions approving of the end in view, and these resolutions have, presumably, been sent to the senators and representatives of the respective States.

As the result of these measures, considerable interest has been excited in Congress in favor of the pending bill, but not enough to secure its passage.

Under these indicated circumstances, it becomes our duty to make still further effort, and we appeal to members of the association everywhere to give us all the help they can. It certainly seems to us that, if the one hundred thousand doctors in the United States would unite as one man and earnestly request of Congress the passage of the bill to create a Department and Secretary of Public Health, the enterprise could not fail of success. Surely, whatever a hundred thousand doctors would ask for would be granted.

We therefore recommend:

1. That every State medical association and every local medical society shall, as promptly as may be, pass resolutions favoring the adoption of our bill, and that such resolutions shall be published in the medical journals, and copies forwarded to the members of Congress.

2. That every doctor in the United States shall address a private letter in advocacy of our bill to the senators and representatives in Congress from their respective States. Every citizen has the right to appeal to his representatives, no matter whether he is acquainted with them personally or not. Can any one believe that, if all the doctors in any State were to unite in soliciting the senators and representatives of said State to pass this bill, such solicitation would not bear good fruit?

3. The medical journals of the country, as a rule, have heretofore given us generous assistance, and we heartily urge upon them a continuance of their efforts in our behalf.

(Signed) JEROME COCHRAN, Chairman, Montgomery, Ala.
C. G. COMEGYS, Cincinnati, Ohio.
N. S. DAVIS, Chicago, Ill.
J. C. CULBERTSON, Cincinnati, Ohio.
LISTON H. MONTGOMERY, Chicago, Ill.
CHARLES DENISON, Denver, Colo.
U. O. B. WINGATE, Milwaukee, Wis.
W. B. ATKINSON, Philadelphia, Pa.

(Medical periodicals favorable to the movement will please copy.)

